# Kinetics of Indigenous Nitrate Reducing Sulfide Oxidizing Activity in Microaerophilic Wastewater Biofilms

**DOI:** 10.1371/journal.pone.0149096

**Published:** 2016-02-12

**Authors:** Desirée Villahermosa, Alfonso Corzo, Emilio Garcia-Robledo, Juan M. González, Sokratis Papaspyrou

**Affiliations:** 1 Departamento de Biología, Facultad de Ciencias del Mar y Ambientales, Universidad de Cádiz, Pol. Río San Pedro s/n, 11510-Puerto Real, Cádiz, Spain; 2 Instituto de Recursos Naturales y Agrobiología, IRNAS-CSIC, Avda. Reina Mercedes 10, 41012-Sevilla, Spain; University of Notre Dame, UNITED STATES

## Abstract

Nitrate decreases sulfide release in wastewater treatment plants (WWTP), but little is known on how it affects the microzonation and kinetics of related microbial processes within the biofilm. The effect of nitrate addition on these properties for sulfate reduction, sulfide oxidation, and oxygen respiration were studied with the use of microelectrodes in microaerophilic wastewater biofilms. Mass balance calaculations and community composition analysis were also performed. At basal WWTP conditions, the biofilm presented a double-layer system. The upper microaerophilic layer (~300 μm) showed low sulfide production (0.31 μmol cm^-3^ h^-1^) and oxygen consumption rates (0.01 μmol cm^-3^ h^-1^). The anoxic lower layer showed high sulfide production (2.7 μmol cm^-3^ h^-1^). Nitrate addition decreased net sulfide production rates, caused by an increase in sulfide oxidation rates (SOR) in the upper layer, rather than an inhibition of sulfate reducing bacteria (SRB). This suggests that the indigenous nitrate reducing-sulfide oxidizing bacteria (NR-SOB) were immediately activated by nitrate. The functional vertical structure of the biofilm changed to a triple-layer system, where the previously upper sulfide-producing layer in the absence of nitrate split into two new layers: 1) an upper sulfide-consuming layer, whose thickness is probably determined by the nitrate penetration depth within the biofilm, and 2) a middle layer producing sulfide at an even higher rate than in the absence of nitrate in some cases. Below these layers, the lower net sulfide-producing layer remained unaffected. Net SOR varied from 0.05 to 0.72 μmol cm^-3^ h^-1^ depending on nitrate and sulfate availability. Addition of low nitrate concentrations likely increased sulfate availability within the biofilm and resulted in an increase of both net sulfate reduction and net sulfide oxidation by overcoming sulfate diffusional limitation from the water phase and the strong coupling between SRB and NR-SOB syntrophic relationship.

## Introduction

Sulfate reducing bacteria (SRB) play an important role in the S and C cycles by coupling the production of H_2_S with the oxidation of organic compounds under anoxic conditions. Sulfide production by SRB is a serious environmental problem (e.g. unpleasant odors, toxicity and metal corrosion) with important economic consequences in several industries (e.g. petrochemical, food, drinking water, wastewater treatment) [1, 2]. In these industries, the production of sulfide often occurs within biofilms growing under microaerophilic or oxic conditions [3–5].

Several methods based on different chemical and biological principles have been proposed during the last decades for mitigating the problems caused by the SRB activity [4]. Among these methods, the addition of nitrate is considered one of the best options to keep sulfide levels under control [5, 6]. In principle, nitrate addition might come about reducing sulfide levels in two ways: 1) by stimulating heterotrophic nitrate reduction to the detriment of sulfate reduction, as nitrate is energetically a more favorable electron acceptor than sulfate. 2) by oxidation of sulfide to elemental sulfur or sulfate by nitrate reducing sulfide oxidizing bacteria (NR-SOB). The metabolism of NR-SOB couples the oxidation of sulfide to the reduction of nitrate all the way to N_2_ under anoxic conditions [7]. This chemolithotrophic metabolism allows establishing a syntrophic ecological relationship between SRB and NR-SOB; SRB supply H_2_S to NR-SOB which in turn oxidize it back to sulfate directly or through elemental sulfur as an intermediate [8].

In wastewater systems, the addition of NO_3_^-^ seems to enhance the activity of NR-SOB [4, 9, 10] with no or little effect on the sulfate reduction rate [5]. Okabe et al. [11] found that the addition of 500 μM NO_3_^−^ during 2 days does not change the SRB community but it does induce the interspecies competition between heterotrophic nitrate reducing bacteria (hNRB) and SRB for common carbon sources. In addition, they observed an increase in the oxidation rate of sulfide that indicates a stimulation of NR-SOB activity as well. Garcia-de-Lomas et al. [5] found that the NR-SOB community was mainly located within the biofilm growing in a waste water treatment plant (WWTP). The addition of NO_3_^-^ induced a quick suppression of net H_2_S production within 2 h. Since microbial growth rates in mature biofilms are typically low or approaching zero [12, 13], the effect of NO_3_^-^ addition occurred by the stimulation of a pre-existent indigenous community of NR-SOB [4, 5]. Recently, nitrate dosing to an experimental sulfide-producing sewer biofilm reactor reduced sulfide and methane production considerably [14].

The existence of an indigenous NR-SOB community in WWTP biofilms opens the possibility of engineering the biofilm’s net metabolism, i.e. to suppress net H_2_S production in the biofilm by the addition of the minimal amount of NO_3_^-^ necessary. The reduction of the NO_3_^-^ dose, and associated costs would likely encourage the application of this environmental technology in the WWTP industry. Nitrate addition to prevent sulfide net production and emission have been used in other industrial sectors and applications, such as the petroleum industry to supress souring and biocorrosion [15], biofiltration of hydrogen sulfide from biogas [16–18] or natural gas [19], and molasses industry [20]; and could be applicable to industrial waste water, for example, from food and fermentation industry, tanneries, kraft pulping, etc. [2]. Sulfide elimination efficiency in the water phase of a pilot scale bioreactor fed directly with wastewater increased with increasing nitrate concentrations following Michaelis–Menten kinetics (K_s_ = 0.63 mM NO_3_^-^ in [6]). After 3 days of NO_3_^-^ dosing, the authors using RNA-based molecular techniques detected an increase of NR-SOB activity in the nitrate amended bioreactor biofilm with respect to the control biofilm [21]. However, no clear relationship was found between the changes in activity of selected NR-SOB species and the amount of nitrate dosed.

The objective of the experiments presented here was to study in detail the kinetics of net sulfide production at a micrometer scale within a biofilm grown in a large-size WWTP, in relation to the availability of sulfate and nitrate in the overlying water phase. H_2_S, pH and O_2_ microelectrodes were used in combination with modeling of concentration profiles and molecular biology techniques (i) to calculate kinetic parameters of indigenous microaerophilic biofilms related to Sulfate Reduction Rate (SRR) and Sulfide Oxidation Rate (SOR); (ii) to localize these processes within the biofilm at a microscale, and (iii) to detect possible changes in the biofilm’s microbial community structure after nitrate dosing.

## Materials and Methods

### Biofilm growth and sampling

Microbial biofilms were grown for eight months on stainless steel coupons (AISI-316L, 15 x 10 cm, n = 5) placed in an aluminium frame in the effluent channel of the Screen Room (Grit Removal) of the Guadalete Waste Water Treatment Plant (Jerez de la Frontera, Spain). Biofilm covered 94–98% of the coupons’ surface and were 2–3 mm thick. Nutrients mean concentrations in the bulk water during biofilm growth were 2.0 mM for ammonia, 4.6 mM for sulfide, 2.7 mM for sulfate, 0.07 mM for nitrate, and 0.01 mM for nitrite. Chemical oxygen demand (COD) was 531 mg/L. For further description of the WWTP conditions see [21]. Each coupon was taken and transferred to the lab within 30 minutes.

### Artificial wastewater

Experiments in the laboratory were done using synthetic wastewater. Its composition was based on the recommendations of Boeije et al. [22] to favor growth of SRB and NR-SOB in flow reactors and complemented with components of the culture media DSMZ 113 and 63 (Table 1). For the 2 and 10 mM sulfate treatments, Na_2_SO_4_ was added to the artificial wastewater to reach those final concentrations. pH was adjusted at 7.0 with HCl or NaOH.

**Table 1:** Composition of basic artificial wastewater (pH 7.0) used in the flow through reactor experiments. All components were the same during all experiments with the exception of Na_2_SO_4_ that was adjusted according the sulfate treatment used.

COMPOUND CATEGORY	COMPOUND	CONCENTRATION
**Organic** (mg L^-1^)	Starch	122.0
	Milk Powder	116.2
	Urea	91.7
	Yeast extract	52.2
	Peptone	17.4
**Inorganic** (mg L^-1^)	Na_2_SO_4_	0^a^, 284.1^b^, or 1420.4 ^c^
	KH_2_PO_4_	234.0
	Na-Ac 3H_2_O	131.6
	NH_4_Cl	127.5
	MgHPO_4_ 3H_2_O	29.0
	NaHCO_3_	25.0
	CaCl_2_	10.0
**Trace compounds** (μg L^-1^)	CoCl_2_ 6H_2_O	50.0
	FeCl_2_ 4H_2_O	41.5
	ZnCl_2_	5.4
	CuCl_2_ 2H_2_O	5.4
	NiCl_2_ 6H_2_O	3.0
	MnCl_2_ 2H_2_O	1.3

^a^ for the experiments at increasing sulfate concentrations.

^b^ to reach 2 mM sulfate final concentration.

^c^ to reach 10 mM sulfate final concentration.

### Set up and operation

Microsensor measurements were performed at 20°C in a plexiglass flow-through chamber (288 mL) [5] under a continuous flow of 40 mL min^-1^ artificial waste water supplied from a 5.8 L reservoir using a peristaltic pump (Watson Marlow cassette multichannel 205S). The artificial wastewater in the reservoir was N_2_-bubbled for at least 20 minutes before the experiment begun to decrease oxygen concentration to about 10 μM. Re-oxygenation of the artificial wastewater during its circulation in the flow chamber (having a very high surface/volume ratio) was reduced by N_2_- bubbling directly in the flow chamber to ensure microaerophilic conditions.

Initial steady state conditions within the biofilm, determined by the recording of two or more equal consecutive profiles, were reached after about two hours. Treatments with different NO_3_^-^ or SO_4_^2-^ concentrations were thereupon started and microprofiles of H_2_S, pH, and O_2_ were measured every 15–20 minutes. Three different artificial wastewater treatments were applied to each coupon/biofilm (n = 5) in the following order: (1) increasing concentrations of SO_4_^2-^ (0, 1.5, 3, 6, 9, and 15 mM of Na_2_SO_4_) without addition of NO_3_^-^, (2) increasing concentrations of NO_3_^-^ (0.15, 0.25, 0.5, 1, 2, and 4 mM of NaNO_3_) at a fixed concentration of 2 mM SO_4_^2-^, and (3) increasing concentrations of NO_3_^-^ (same as before) at a fixed concentration of 10 mM SO_4_^2-^. SO_4_^2-^ and NO_3_^-^ were added to the artificial wastewater reservoir bottle from stock solutions only once steady state was achieved with the previous concentration. For each concentration a new reservoir bottle was used.

### Microsensor measurements and calculations

Vertical microprofiles of O_2_, pH and H_2_S were measured in the biofilms using microelectrodes (tip diameter 25 to 50 μm, Unisense®). The microelectrodes, connected to a microsensor Multimeter, were mounted on a computer controlled micromanipulator (MC-232, Unisense®) and driven down into the biofilms with a step resolution of 100 μm. The profiles were done using the control program Sensor TracePRO (Unisense®). The oxygen microelectrode [23] was calibrated using 100% O_2_ saturated artificial wastewater and anoxic wastewater (N_2_ bubbled). The conversion from saturation to concentration was done applying the saturation values provided by Ramsing and Gundersen (Unisense manuals). The pH microelectrode was calibrated using commercial pH buffers of pH 4.0 and 7.0. The H_2_S microelectrode [24] was calibrated by the addition of known volumes of a concentrated H_2_S stock (50 mM) into an anoxic pH 4.0 buffer solution.

Net consumption and production rates as a function of depth were estimated by modeling the H_2_S and O_2_ profiles with the numerical model developed by Berg et al. [25]. The model allows determining the vertical location of production and consumption inside the biofilm at a microscale assuming steady state conditions for the concentrations measured, and taking into account molecular diffusion. The model uses a least square fit and F-testing model that reproduces a concentration profile with the simplest production-consumption profile. Diffusivity in water (D) used for the profile modeling was 1.32x 10^−5^ cm^2^ s^-1^ for H_2_S and 1.75 x 10^−5^ cm^2^ s^-1^ for O_2_ at 20°C, calculated from the values at 25°C using the Stokes-Einstein equation [26]. Molecular diffusion in the biofilm (D_s_) for H_2_S and O_2_ were calculated according to a relative diffusivity in biofilms (D_s_/D) of 0.6 [27].

Maximum penetration in the biofilm of a given substrate (z_max_) can be calculated from the concentration of the compound in the bulk water phase, the volumetric consumption rate within the biofilm and the effective diffusive boundary layer (DBL) according to Eq 1 [28],

$$z_{max}={\left(\frac{2C_{0}D_{s}}{R}\right)}^{1/2}$$ (1)

where C_o_ is the concentration at the biofilm surface, D_s_ is the apparent diffusion coefficient within the biofilm and R is the volumetric consumption of the compound within the biofilm. C_o_ was calculated for NO_3_^-^ and SO_4_^2-^ from their bulk water phase concentrations according to Eq 2 for zero order kinetics [28, 29],

$$C_{o}=C_{w}+\left(\frac{z_{\delta }^{2}}{D^{2}}\right)\times RD_{S}\times \left(1-\sqrt{\left(\frac{2D^{2}C_{w}}{RD_{s}z_{\delta }^{2}}+1\right)}\right)$$ (2)

where C_w_ is the concentration in the mixed, overlying bulk water phase and z_δ_ is the thickness of the effective DBL [30]. The value of z_δ_ for sulfate and nitrate were calculated from z_δ_ for oxygen, which was directly determined from measured O_2_ profiles [30]. Penetration depth of sulfate and nitrate within the biofilm at 20°C were calculated using D of 0.952 x10^-5^ cm^2^ s^-1^ and 1.23 x 10^−5^ cm^2^ s^-1^ respectively calculated using the Stokes-Einstein equation from values at 25°C [26, 31]. The effective molecular diffusion coefficients in the biofilm (D_s_) for sulfate and nitrate were calculated according to a relative diffusivity in biofilms (D_s_/D) for inorganic anions and cations of 0.6 [27].

### Water phase monitoring

Water samples for analysis of NO_3_^-^, NO_2_^-^, NH_4_^+^ and SO_4_^2-^ were taken from the influent and effluent of the flow-through chamber after steady state conditions were reached in every treatment. Samples were immediately filtered through pre-combustioned GF/F filters (0.7 μm nominal pore size) and kept frozen until analyses. NO_3_^-^ and NO_2_^-^ concentrations were determined photometrically after García-Robledo et al. [32]. NH_4_^+^ was determined after Bower and Holm-Hansen [33]. SO_4_^2-^ samples were analyzed by turbidimetry after APHA [34].

### Microbial community composition

Samples to analyze changes in the microbial community during the experiments were taken before the initiation of the treatments (after steady state was achieved in the flow-through chamber) and 10–13 h after initiating sulfate or nitrate additions. DGGE of the microbial community profiles before and after the treatments were performed with cDNA as previously described [35]. Collected samples were preserved with RNA-later® (Ambion, Inc.) (10x volume) and frozen at -80°C in the laboratory. RNA was extracted as in [36]. The cDNA was generated from the extracted RNA and the reverse transcription reaction was performed with ThermoScript (Invitrogen, Carlsbag, CA) 60 sec at 55°C and 5 sec at 85°C using primer 518r [37]. Amplification by PCR of fragments of the bacterial 16S ribosomal RNA genes was performed with the primer pair 341f-GC and 518r [37] under the following cycling conditions: 94°C for 3 min; 40 cycles of 94°C for 20 s, 55°C for 20 s, 72°C for 1 min and 72°C for 30 min. Bacterial community fingerprints were carried out by denaturing gradient gel electrophoresis (DGGE) as described in [37]. Significance of differences between bacterial community fingerprints was analyzed as previously described [38].

### Statistical methods

Values in tables and figures are given as means ± standard deviations in all cases. Signed Rank test for statistical analysis was performed with SigmaPlot 11.0 (Systat Software, Inc).

## Results

### Oxygen, sulfide and pH microprofiles and biofilm microzonation

**Fig 1:**
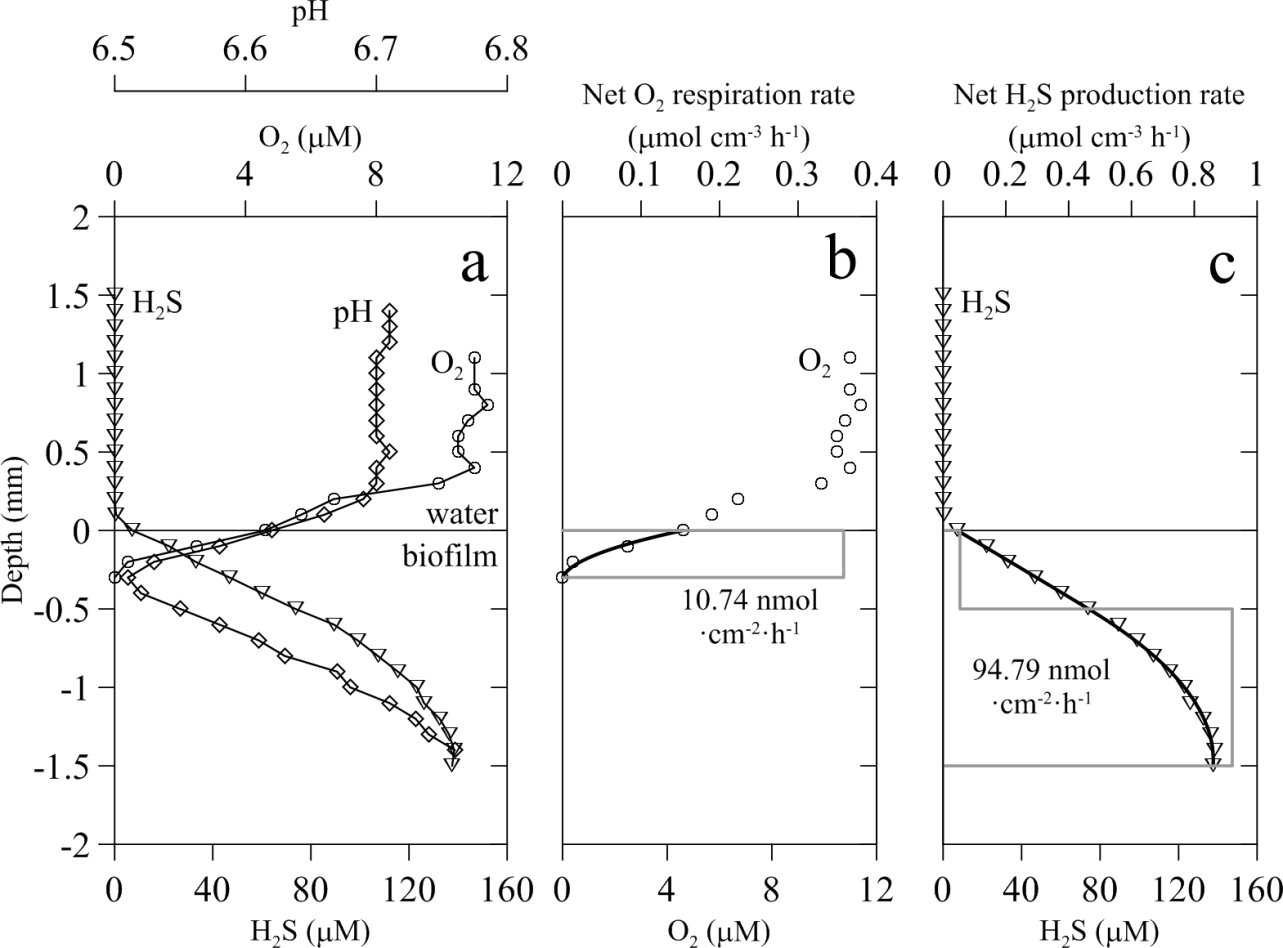
Vertical H_2_S, O_2_ and pH profiles in wastewater biofilm and modeled reaction rates. (a) Representative H_2_S (triangles), O_2_ (circles) and pH (diamonds) profiles in a biofilm at 1.5 mM sulfate and no nitrate; (b) and (c) modeled concentration profile (thick black lines) and volumetric reaction rates (grey straight lines) for O_2_ and H_2_S, respectively. Depths with the same rates determine the biofilm microzonation. Areal rates of O_2_ consumption and net sulfide production are also indicated (nmol cm^-2^ h^-1^).

Biofilms were maintained under microaerophilic conditions during all the experiments, with the mean oxygen concentration in the bulk water at basal WWTP conditions (1.5–3 mM sulfate) being around 10.1 ± 12.3 μM (n = 20). Oxygen was quickly consumed within the first 300 μm into the biofilms creating a narrow microaerophilic layer within the biofilms (Fig 1). Mean oxygen concentration and volumetric consumption rate, calculated by numerical modeling of O_2_ profiles, were 3 μM O_2_ and 0.31 ± 0.3 μmol O_2_ cm^-3^ h^-1^, respectively. Mean depth integrated respiration in this upper layer of the biofilms was 0.010 ± 0.009 μmol O_2_ cm^-2^ h^-1^.

**Fig 2:**
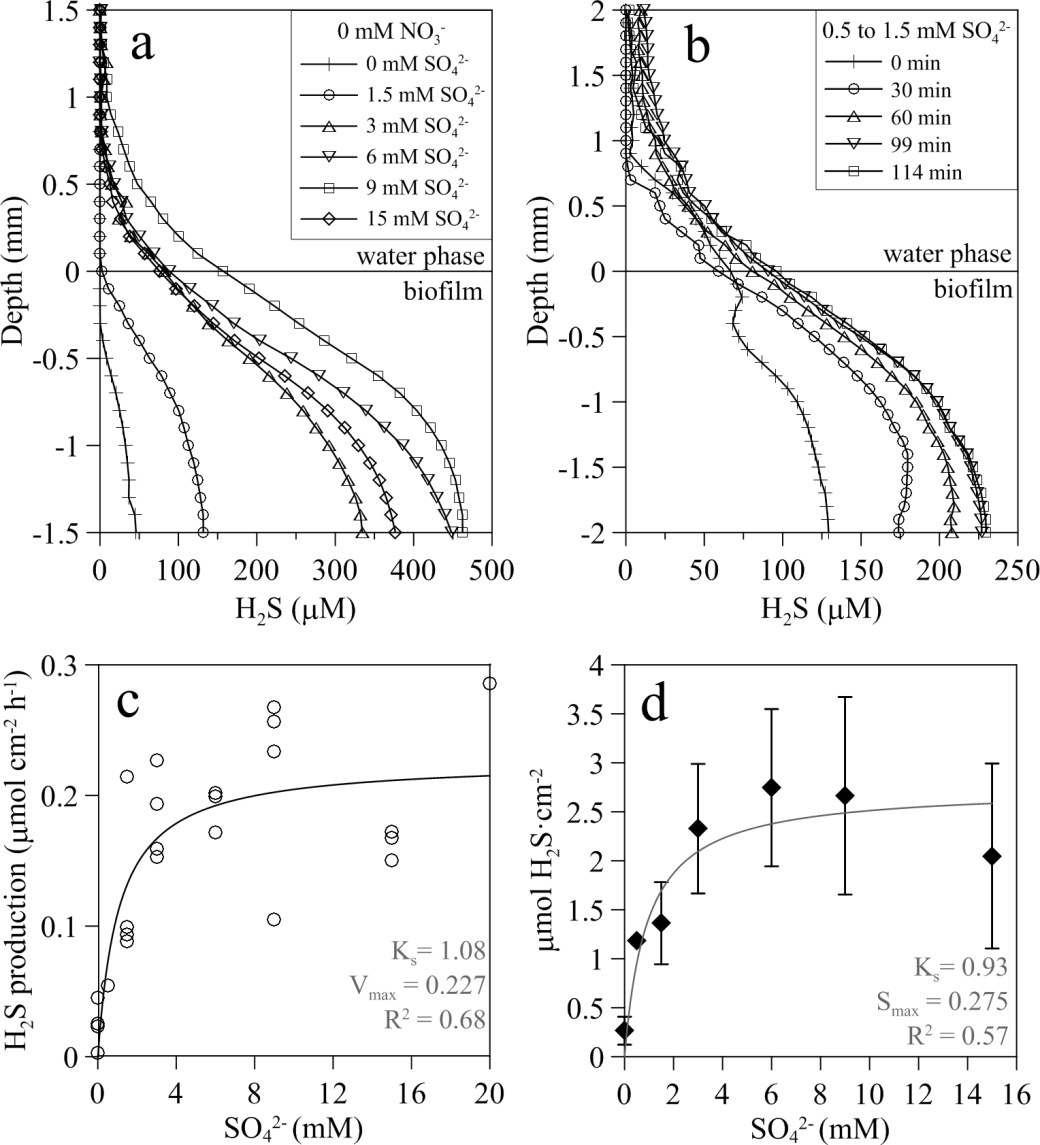
Sulfide dynamics in wastewater biofilms. (a) Representative vertical H_2_S concentration profiles with increasing sulfate concentrations. (b) Example of evolution of sulfide concentration profiles over time in a biofilm subjected to an increase of sulfate concentration from 0.5 to 1.5 mM. (c) Net areal sulfide production (first 1.5 mm of the biofilm) with increasing sulfate concentrations. (d) Mean H_2_S concentration integrated in depth (μmol cm^-2^) ± standard deviation with increasing concentrations of sulfate (n = 1 for 0.5 and 20 mM SO_4_^2-^, n = 4 for 6 and 15 mM, n = 5 for the rest). Integrated mean H_2_S concentrations were calculated from measured H_2_S profile from the surface up to a depth of 1.5 mm within the biofilm.

In the absence of nitrate, biofilms used in the experiments were always net sulfide producers. Despite the presence of small amounts of oxygen in the upper biofilm layer, sulfide was detected and released to the water phase at a rate of 0.21 ± 0.13 μmol cm^-2^ h^-1^. Sulfide concentration increased progressively with depth, with the maximum values being measured at the deepest layer at 1.5–2.0 mm depth (Figs 1; 2A and 2B). Numerical modeling of sulfide profiles usually detected two layers. The upper layer had a similar thickness to that of the microaerophilic oxic layer and showed lower sulfide production rates of 0.06 ± 0.10 μmol cm^-3^ h^-1^ (see example of profile in Fig 1) compared to the deepest biofilm layer, which was characterized by high sulfide production rates of 2.7 ± 3.1 μmol cm^-3^ h^-1^.

Profiles of pH showed a decrease (about 0.2 units) at the biofilm-water interface, in parallel to the decrease of oxygen concentrations in the upper biofilm layer. Once O_2_ was exhausted, pH increased progressively with depth (Fig 1).

### Effects of sulfate

Steady state sulfide concentrations inside the biofilm were clearly dependent on sulfate availability in the overlying water phase (Fig 2A). Sulfide production and oxidation processes responded very quickly to changes in the concentration of sulfate in the bulk water phase (Fig 2B). The largest changes occurred within the first 30 min (Fig 2B). Profiles reached a new steady state within 90 min, regardless of the concentration assayed. Net sulfate reduction rate (or net sulfide production rate), calculated by modeling the measured sulfide profiles, increased with water phase sulfate concentrations following a Michaelis-Menten kinetics (K_s_ = 1.08 ± 0.53 mM SO_4_^2-^, maximum integrated net sulfate reduction = 0.23 ± 0.02 μmol cm^-2^ h^-1^, r^2^ = 0.687, Fig 2C). Similarly, the relation of integrated sulfide concentration within the biofilm with water phase sulfate seemed to follow a saturating kinetics (K_s_ = 0.87 mM SO_4_^2-^, maximum sulfide integrated concentration = 2.70 μmol cm^-2^, r^2^ = 0.567, Fig 2D). At very high sulfate concentrations (≥15 mM), sulfide production was saturated and integrated sulfide concentration within the biofilm diminished (Fig 2A and 2D).

**Fig 3:**
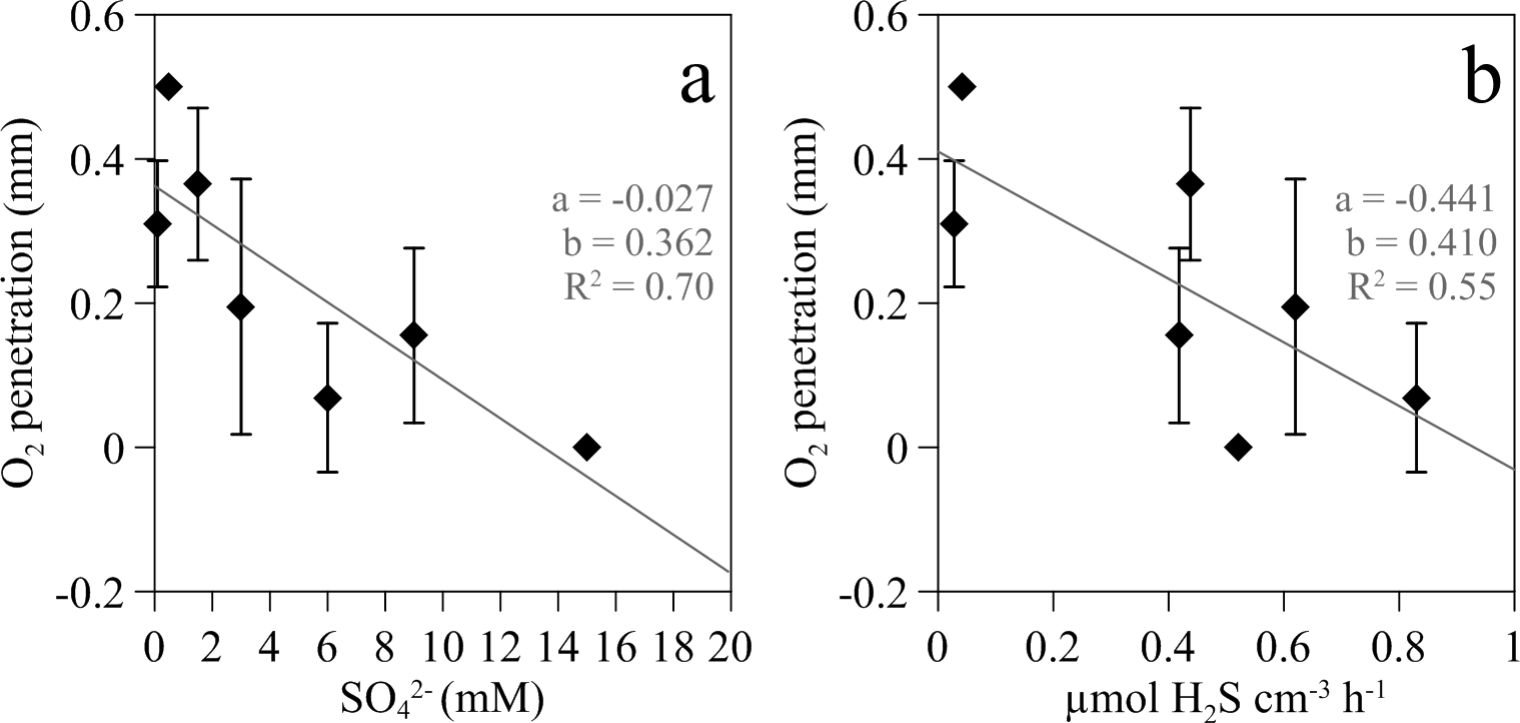
Maximum oxygen penetration depth in wastewater biofilms. (a) Maximum oxygen penetration depth (Zox) in the biofilm under different sulfate concentrations in the bulk water phase and (b) Maximum oxygen penetration depth (Zox) in the biofilm as a function of sulfide production rate in the upper production layer. Bars represent standard deviation of means (O2 penetration depth from n = 2 (penetration values at 0.5 and 20 mM), 8 (values at 6 and 15 mM) or 10 (the rest) profiles.). Inserted in every plot is the slope of the regression line (a), the intercept (b) and determination coefficient (R2).

Sulfate availability affected considerably the penetration of oxygen into the biofilm. Despite large variability between biofilms, maximum oxygen depth penetration into the biofilm (z_O_) decreased linearly as sulfate concentration and net sulfide production rate increased (Fig 3).

### Effects of nitrate

**Fig 4:**
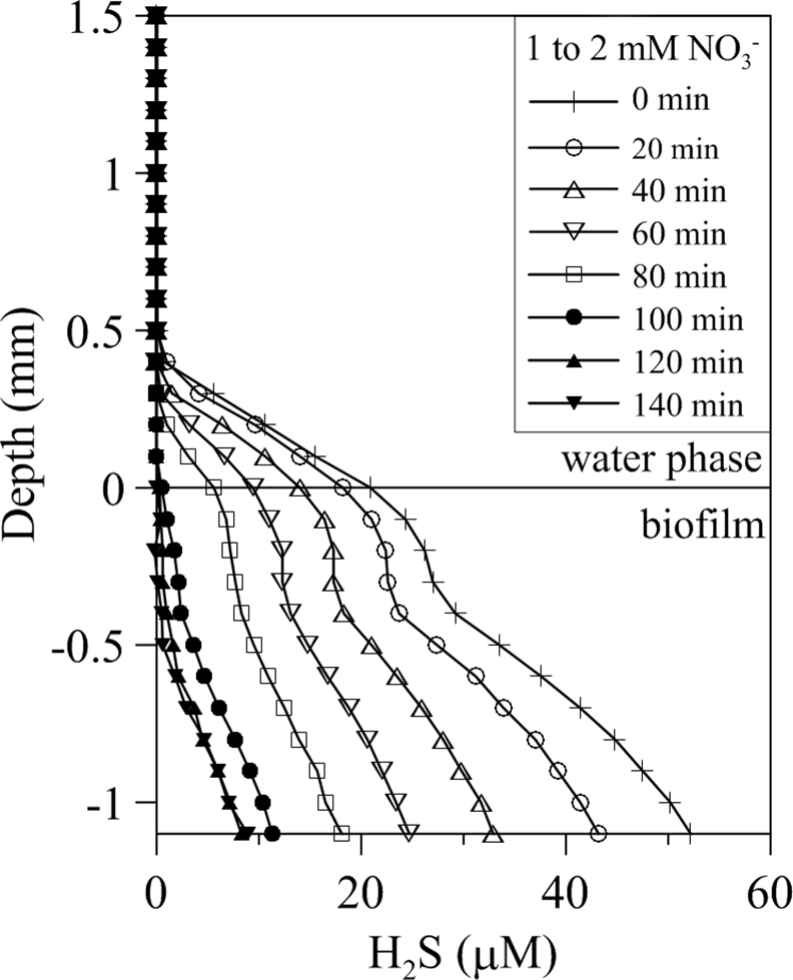
Temporal evolution of vertical H_2_S profiles in wastewater biofilms amended with nitrate. Representative temporal evolution of H_2_S profiles in wastewater biofilms adapting to an increase of nitrate concentration from 1 mM to 2 mM at a fixed 2 mM sulfate concentration.

**Fig 5:**
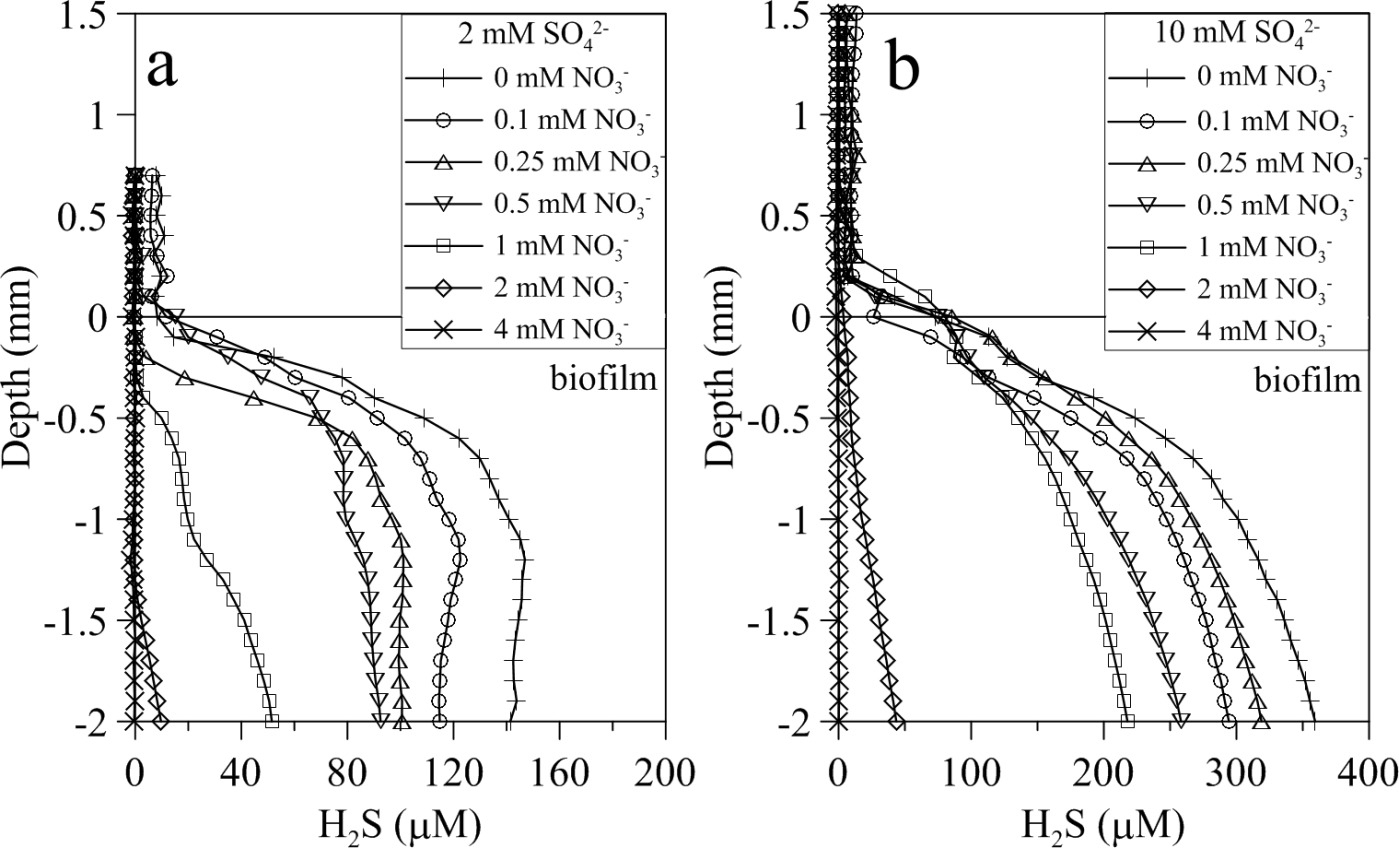
Vertical microprofiles of H_2_S in wastewater biofilms amended with increasing nitrate concetrations. Representative steady state H_2_S profiles **in wastewater biofilms** following the addition of increasing nitrate concentrations (from 0 to 4 mM nitrate) at (A) 2 mM sulfate and (B) 10 mM sulfate.

**Fig 6:**
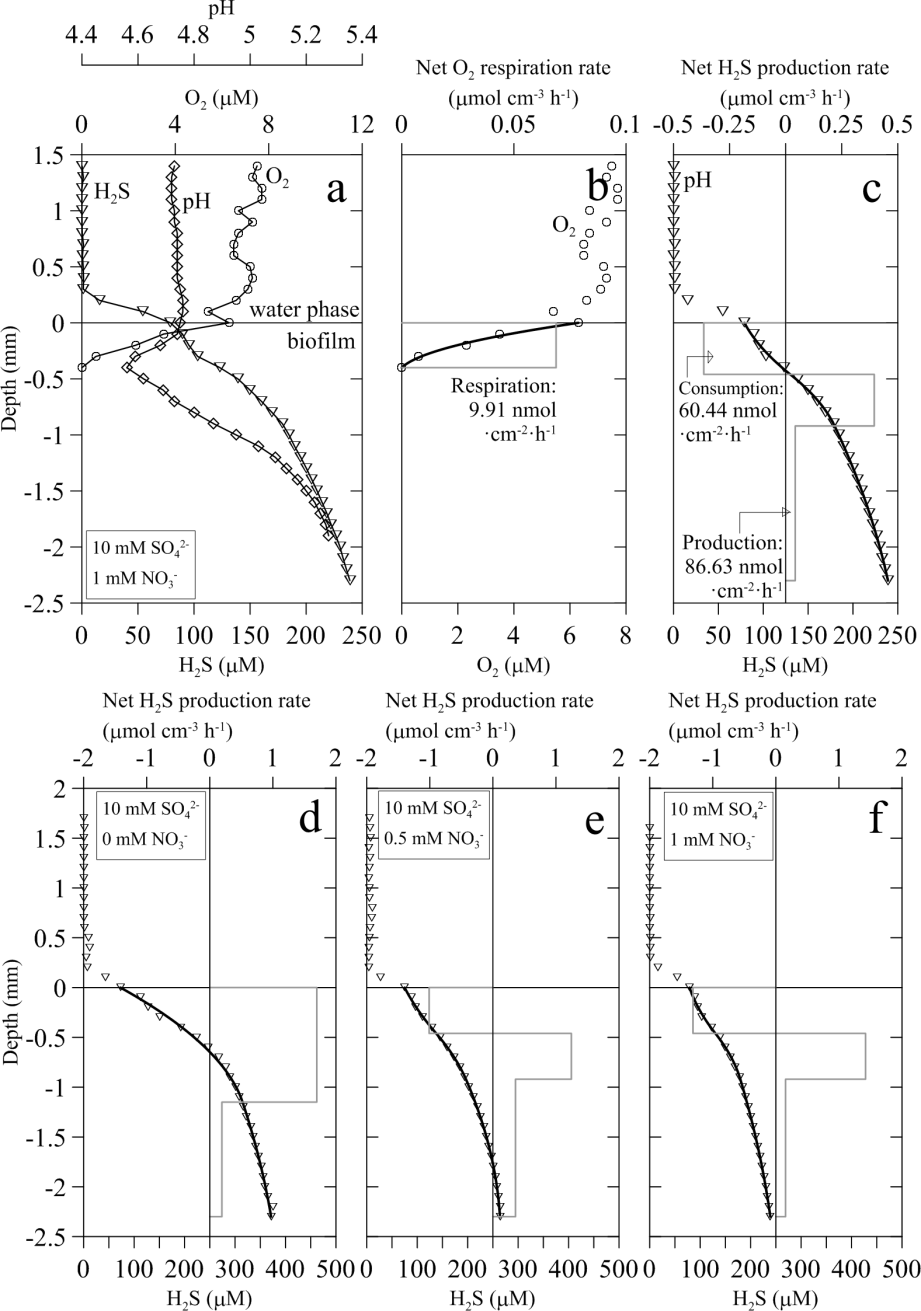
Vertical microprofiles of H_2_S, O_2_ and pH in wastewater biofilms amended with sulfate and nitrate. (A) Representative H_2_S (triangles, O_2_ (circles) and pH (diamonds) profiles in a biofilm with 10 mM sulfate and 1 mM nitrate and modeled profiles of (B) O_2_ and (C) H_2_S. Real data represented with symbols and modeled profiles with bold black lines. Boxes (grey lines) represent volumetric respiration and sulfide production profiles (μmol cm^-3^ h^-1^). Depths with the same rates determine the biofilm microzonation. (B) Areal rates of O_2_ consumption and (C) net sulfide production (nmol cm^-2^ h^-1^). (D, E and F) Changes in H_2_S concentration, modeled profiles and volumetric net sulfide production rates with depth are shown in lower panels at 10 mM sulfate and increasing nitrate concentrations from 0 to 1 mM nitrate.

The addition of nitrate to the bulk water phase induced a progressive decrease of sulfide concentration within the biofilm over time as shown by the successive sulfide profiles (Fig 4). Sulfide profiles reached a new steady state before 120 min. This nitrate-dependent sulfide decrease occurred at both subsaturating and saturating sulfate concentrations (2 and 10 mM SO_4_^-2^, respectively) (Fig 5A and 5B). However, when sulfate was provided at saturating concentration, higher amounts of nitrate were needed to fully suppress the release of sulfide to the water column (Fig 5B). Addition of nitrate, on the other hand, did not induce perceptible changes to the general shape of O_2_ and pH vertical profiles (Figs 1A and 6A). Numerical modeling of H_2_S profiles in the presence of added nitrate showed a net sulfide consumption in the upper 500 μm of the biofilm, attributed to increased sulfide oxidation at this layer (Fig 6C, 6E and 6F). Net sulfide oxidation rate in this upper layer increased with nitrate concentration (Fig 6D, 6E and 6F). Deeper in the biofilm, net sulfide production rate was observed with highest values at the 500–1000 μm layer.

**Fig 7:**
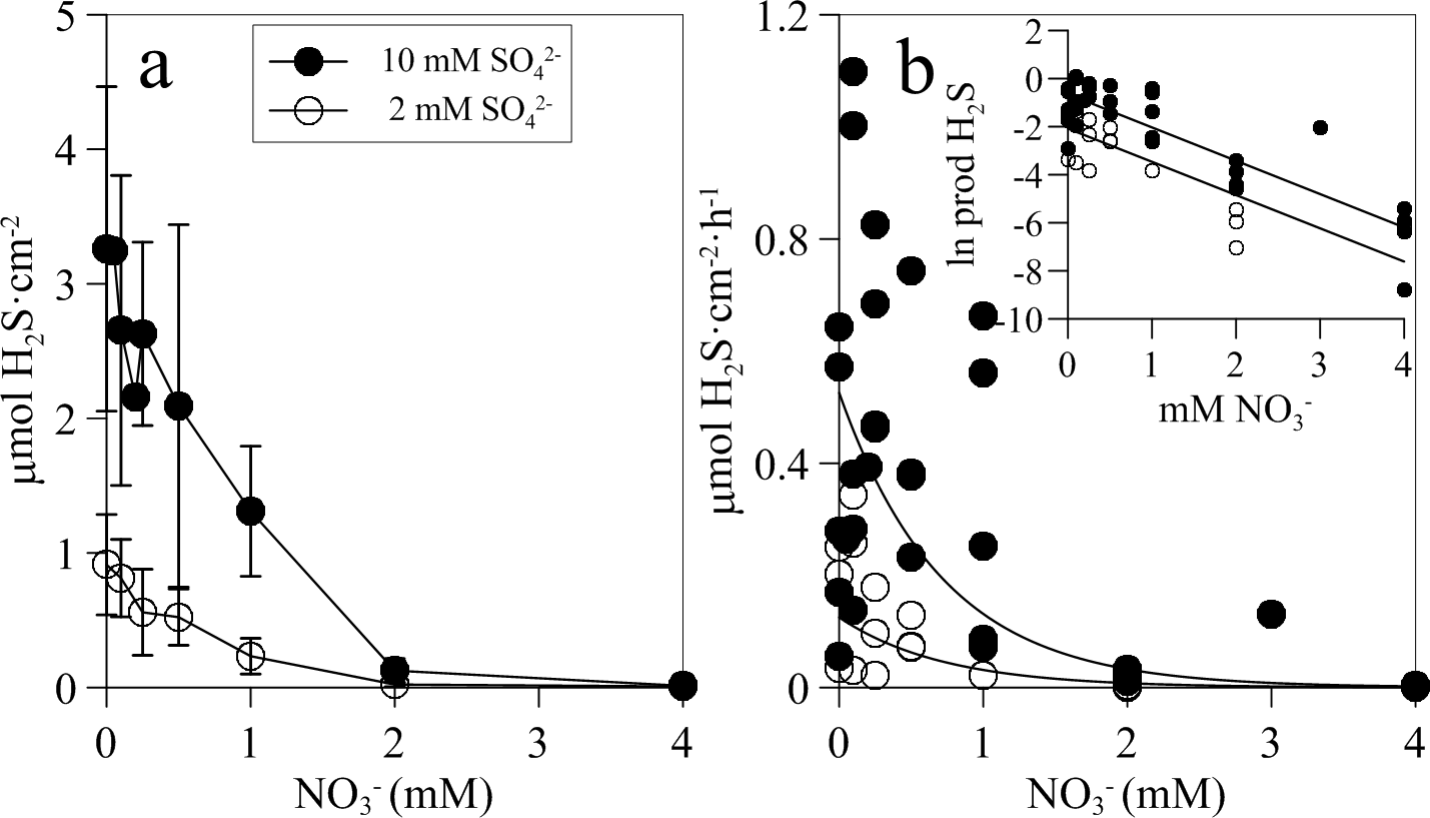
Relation of sulphide concetration and depth-integrated net sulphide production rates in wasterwater biofilms amended with different concentrations of nitrate (0–4 mM). (a) Mean H_2_S concentration integrated in depth (μmol cm^-2^) ± standard deviation and (b) depth-integrated net H_2_S production rates at different concentrations of nitrate. Inserted graph presents the same data fitted to a linearized exponential decay equation (y = a e^−b x^, ln y = ln a - bx), where b = -1.3840, ln a = -2.08, R^2^ = 0.65 (at 2 mM sulfate) and b = -1.3869, ln a = -0.64 and R^2^ = 0.75 (at 10 mM sulfate) (p values < 0.0001).

The effect of nitrate addition on sulfide was concentration dependent, wtih the average integrated sulfide concentration within the biofilm decreasing as added nitrate increased (Fig 7A). Addition of 2 mM nitrate reduced the integrated sulfide concentration within the biofilms to as low values as 0.22 ± 0.17 μmol H_2_S cm^-2^ and 1.26 ± 0.89 μmol H_2_S cm^-2^ in the presence of 2 mM and 10 mM sulfate, respectively. Similarly, aerial net sulfide production rate for the biofilm decreased exponentially as nitrate concentration increased following a first order kinetics with the same slope at both 2 and 10 mM sulfate (Fig 7B).

**Fig 8:**
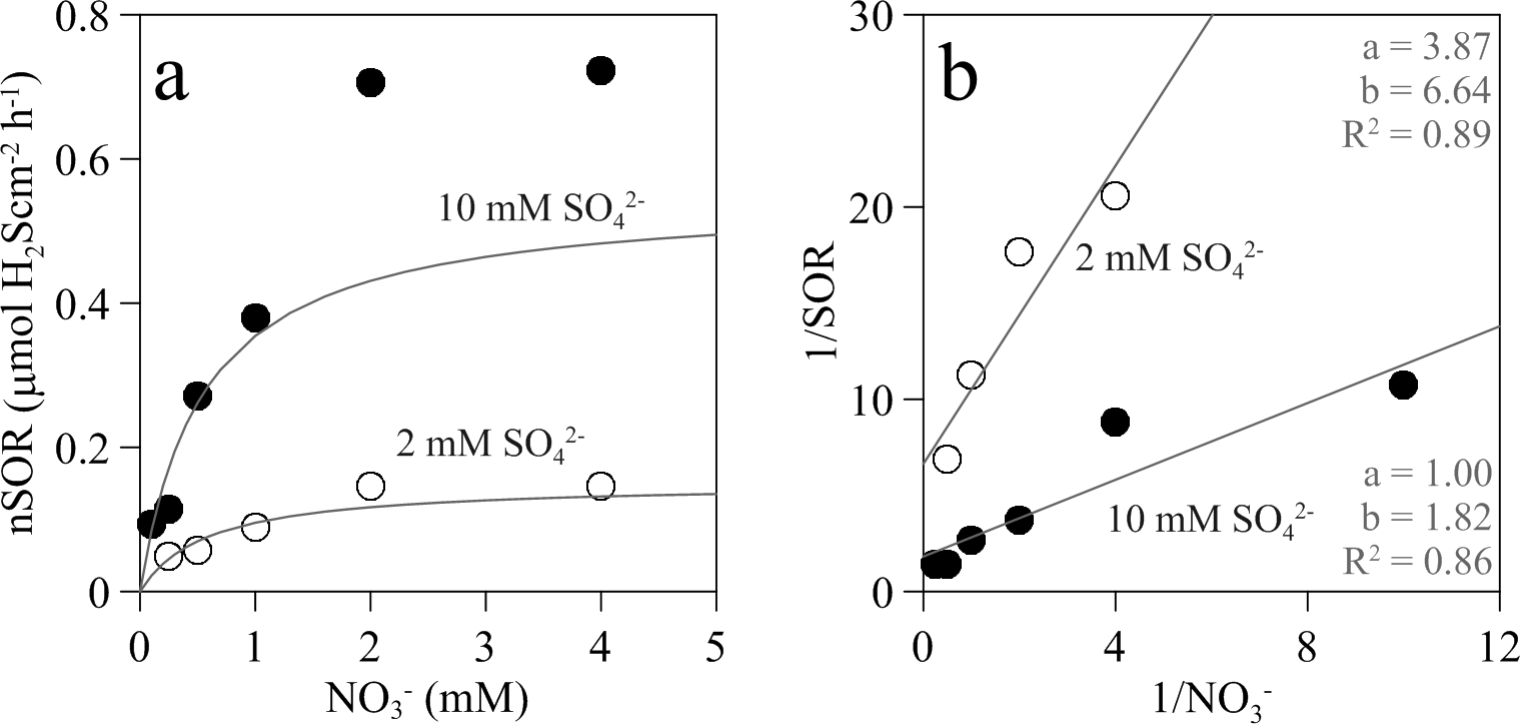
Net sulfide oxidation rate (nSOR) *vs*. added nitrate concentration kinetics in wastewater biofilms amended with different concentrations of sulfate and nitrate. (a) Net sulfide oxidation rate (nSOR) *vs*. added nitrate concentration at fixed concentration of 2 mM and 10 mM sulfate. (b) nSOR was calculated according to Eq. 4. Solid lines are the modeled values calculated from kinetic parameters determined from double inverse plots. Kinetic parameters for the 2 mM sulfate experiment were: K_s_ = 0.58 mM NO_3_^-^, V_max_ = 0.15 mM H_2_S cm^-2^ h^-1^ and for 10 mM sulfate were: K_s_ = 0.55 mM NO_3_^-^, V_max_ = 0.55 mM H_2_S cm^-2^ h^-1^.). Inserted in plot b are the slopes of the regression lines (a), the intercepts (b) and determination coefficients (R^2^).

Assuming that the net sulfate reduction rate, estimated here by the net sulfide production rate in the absence of added nitrate (nSRR_o_), is not affected by the addition of nitrate, net sulfide oxidation rate at different nitrate concentrations (nSOR_N_) can be estimated according to Eq 3,

$$nSOR_{N}=nSRR_{o}-nSRR_{N}$$ (3)

where nSRR_N_ is the net sulfide production rate at a given nitrate addition concentration. Therefore, the maximum nSOR_N_ will be numerically equal to the maximum nSRR_o_ and the relationship of nSOR_N_ with nitrate concentration will be the reciprocal of the exponential decrease of net sulfide production rate with nitrate (Fig 7B). To account for the variability between biofilms, maximum nSOR_N_ was estimated from the decay constant (a) of the exponential decrease equation fitted to the net sulfide production rate at 2 and 10 mM sulfate. Thus, maximum nSOR_N_ was estimated to be 0.148 and 0.724 μmol H_2_S m^-2^ h^-1^, respectively (Fig 7). nSOR_N_ calculated for the various nitrate additions at the two sulfate concentrations fitted significantly (p < 0.05) to a monosubstrate Michaelis-Menten kinetics. Estimated K_s_ values for nitrate were similar for both sulfate concentrations tested, i.e. 0.58 and 0.55 mM for 2 and 10 mM sulfate, respectively. Despite the overall good fit, the calculated maximum nSOR_N_ for the 10 mM sulfate concentration (V_max_ = 0.549 μmol H_2_S m^-2^ h^-1^) was significantly lower than the experimental values measured (Fig 8).

### Nitrate, nitrite, ammonium and sulfate mass balances

**Fig 9:**
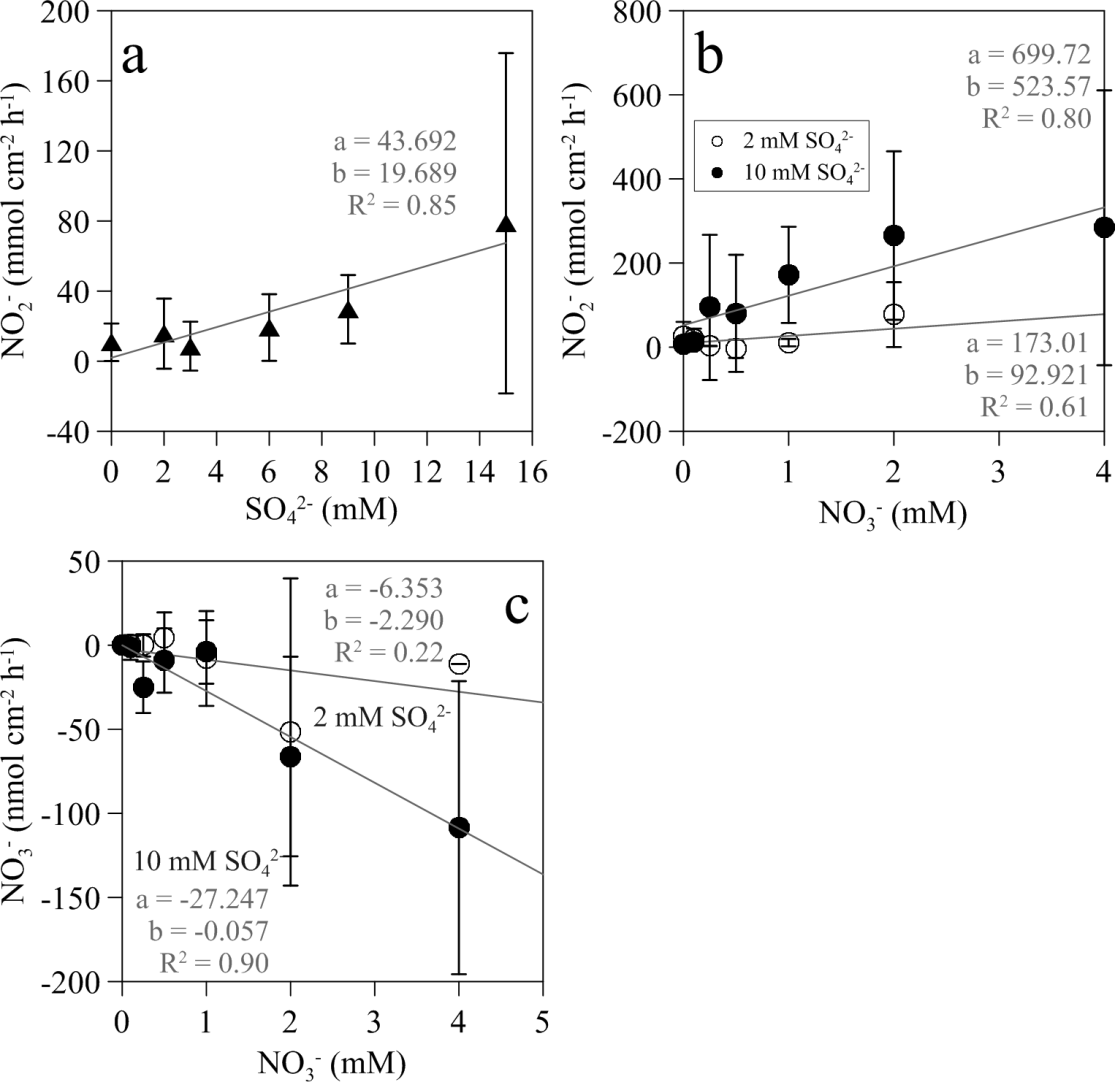
Net mass balance production rates of nitrite and nitrate in wastewater biofilms amended with varying concetrations of sulfate and nitrate. (a) Relation between the nitrite production by the biofilm and increasing sulfate concentration in the bulk water and no added nitrate and (b) on increasing nitrate concentrations in the presence of 2 and 10 mM sulfate. (c) Relation between nitrate production by the biofilm and increasing nitrate concentrations in the presence of 2 and 10 mM sulfate. Inserted in the plots are the slopes of the regression lines (a), the intercepts (b) and determination coefficients (R^2^).

Nitrate, nitrite, ammonium and sulfate net production rates were measured from the difference in concentration between the inflow and outflow of the flow-through incubation chamber. Except for nitrite, there was no strong evidence for dependence between net production rates for these compounds and either increasing sulfate or nitrate concentrations for individual biofilms. Nitrate was consistently consumed in the biofilm only in the experiments at 10 mM sulfate. Ammonia was produced only in the series of incubations at increasing sulfate concentrations in absence of added nitrate (P < 0.001), but showed no consistent differences with nitrate addition. Sulfate inflow and outflow concentrations were not significantly different in the treatments with increasing concentrations of sulfate without added nitrate. In contrast, addition of nitrate clearly stimulated the consumption of sulfate (P = 0.017). Nitrite was consistently produced by the biofilm in all treatments (Table 2). In addition, the net nitrite production rate was linearly dependent on both sulfate and nitrate concentrations, with the maximum rate of net nitrite production observed with 10 mM sulfate and 2–3 mM nitrate (Fig 9A and 9B).

**Table 2:** Mass balance rates for nitrate, nitrite, ammonium and sulfate (nmol cm^-2^ h^-1^). Statistical significance (p value included in the table) of differences in concentrations before and after the flow-through chamber were tested by Signed Rank test during the different incubations: increasing amount of sulfate and no added nitrate (Δ SO_4_^2—^, Δ0 NO_3_^-^, n = 22), increasing amount of nitrate in presence of 2 mM sulfate (2 mM SO_4_^2-^, Δ NO_3_^-^, n = 17) and 10 mM sulfate (10 mM SO_4_^2-^, Δ NO_3_^-^, n = 24). Values are the mean of the difference ± SD of the experiments at variable sulfate or nitrate concentrations. Positive and negative values represent respectively net production and net consumption in the biofilm. Marked with an asterisk are rates with a p value below 0.05.

	ΔSO_4_^2-^, 0 NO_3_^-^		2 MM SO_4_^2-^, Δ NO_3_^-^		10 MM SO_4_^2-^, Δ NO_3_^-^	
	nmol cm^-2^ h^-1^	p value	nmol cm^-2^ h^-1^	p value	nmol cm^-2^ h^-1^	p value
NO_3_^-^	-0.88 ± 0.44	0.500	-8.9 ± 32.8	0.900	-35.0 ± 52.5*	0.007
NO_2_^-^	25.5 ± 39.3*	< 0.001	24.7 ± 41.7*	0.001	126.9 ± 173.4*	< 0.001
NH_4_^+^	7.0 ± 10.5*	0.003	-3.7 ± 14.1	0.579	-1.2 ± 11.9	0.721
SO_4_^2-^	5.0 ± 18.1	0.374	-4.5 ± 6.3*	0.020	-28.9 ± 82.6*	0.017

### Microbial community

**Fig 10:**
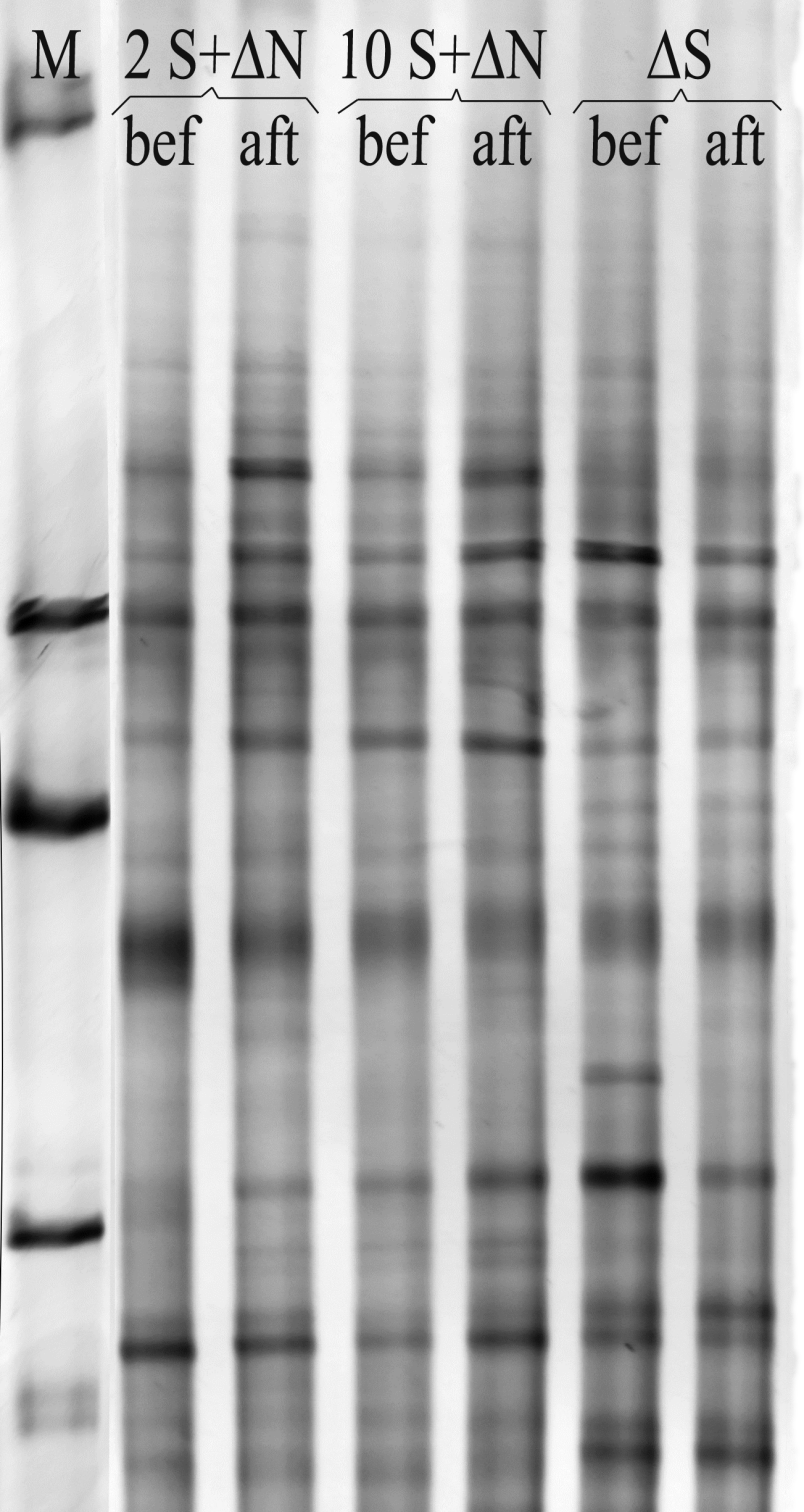
Microbial community DGGE profiles for cDNA extracted from waster water biofilms before and after addition of varying concentrations of sulfate and nitrate. Samples are from biofilms incubated with 2 mM sulfate and increasing amounts of nitrate (2 + ΔN), 10 mM sulfate and increasing amount of nitrate (10 + ΔN), and increasing amount of sulfate and no addition of nitrate to the artificial wastewater (ΔS). Samples were collected before (bef) and 12 hours after (aft) the beginning of nitrate or sulfate addition. Column M represents the marker.

Exposure of the biofilm to different sulfate or nitrate concentrations did not result in noticeable changes in the fingerprints of the dominant, metabolically active, members obacterial communities carried out from cDNA (Fig 10). No significant difference was observed before and after 10–13 h of treatment when comparing cDNA fingerprints of the whole bacterial communities (Fig 10). These fingerprints depict the dominant metabolically active members of the microbial communities suggesting that separated treatments for the course of the experiments do not cause major changes in these communities, even when exposed to different concentrations of sulfate and/or nitrate.

## Discussion

The study presented here shows that at the biofilm scale inhibition of net sulfide production in response to changes in nitrate availability began immediately (Fig 4). This suggests that inhibition of net sulfide production occurs through the activation of a pre-existing metabolic pathway in the biofilms (NR-SOB activity) rather than a change in the composition of the microbial community. Metabolic changes in these biofilms induced by sulfate and nitrate amendments were not reflected as major changes in the wastewater biofilm bacterial communities as confirmed by 16S cDNA fingerprinting (Fig 10). The most abundant members of the bacterial community in WWTP showed low variability in waste water biofilms [4, 39]. Previous works in bioreactors and whole-plant experiments have also shown that suppression of net sulfide production by nitrate addition in wastewater environments occurs quickly, in less than 2–3 hours, and that the effect is reversible in a similar time scale, once nitrate addition is terminated [4, 5].

### Functional microstructure of wastewater biofilms

Wastewater biofilms are complex multispecies stratified communities where many different microbial processes occur simultaneously in the different physicochemical microenvironments created within the biofilm [40]. The microscale information provided by O_2_, H_2_S and pH microsensors revealed that in the absence of added NO_3_^-^, the microaerophilic biofilm was a two-layer system (Fig 1). In the upper layer, O_2_ penetrated down to 0.3–0.4 mm, similar to other wastewater biofilms under either aerobic or microaerophilic conditions [41–44]. However, the O_2_ consumption rate measured in our experiments at a concentration of 0.5–1 mM sulfate was about one order of magnitude lower than those measured under aerobic conditions (0.010 *vs*. 0.24–0.39 μmol O_2_ cm^-2^ h^-1^ and 0.309 μmol O_2_ cm^-3^ h^-1^ *vs*.11-19 μmol O_2_ cm^-3^ h^-1^) [45, 46]. Oxic respiration of organic compounds and the biotic or abiotic re-oxidation of reduced inorganic compounds can contribute to the oxygen consumption in the upper biofilm layer. In our experimental system, the primary oxygen consumption pathway was most likely aerobic biological oxidation of H_2_S, as suggested by the decrease in O_2_ penetration depth as sulfide production rates increased (Fig 3) and by stoichiometric calculations. Given the net sulfide production rate calculated from H_2_S profiles (0.202 ± 0.073 μmol cm^-2^ h^-1^) and assuming complete oxidation of H_2_S to SO_4_^2-^ (i. e. 2 mol of O_2_ consumed 1 mol H_2_S oxidized), the complete oxidation of all the H_2_S would require an oxygen consumption rate of 0.404 μmol O_2_ cm^-2^ h^-1^; that is more than one order of magnitude higher than the actual oxygen consumption rate measured. Oxic sulfide oxidation in biofilms is considered almost entirely a microbial driven process due the slow rate of the chemical oxidation, with a turnover time in the range of half an hour to several hours [47–49]. This was confirmed in our microaerophilic biofilms since with 10 mM sulfate in the bulk water phase, turnover times for O_2_ and H_2_S were of 30–50 s and 300 s respectively. Turnover times in aerobic biofilms growing in a trickling filter were even 1 to 2 orders of magnitude lower [45]. In addition, another characteristic of bacterial mediated sulfide oxidation is its occurrence in both biofilms and marine sediments within a very narrow reaction zone [46] such as that found in this study (Fig 6).

Biogeochemical numerical modeling of H_2_S vertical profiles under typical WWTP sulfate concentrations (1.5–3 mM) confirmed the two-layer functional structure: an upper layer with positive but low net sulfide production rates under microaerophilic conditions (0.06 μmol cm^-3^ h-1) and a deeper layer extending to the bottom of the biofilm with high net sulfide production rates (2.7 ± 3.1 μmol cm^-3^ h^-1^). However, the experimental increase of sulfate concentration, in the absence of added nitrate, induced an increase of net sulfide production rates and the expansion of the upper biofilm layer down to 1 mm depth. However, net sulfide production rate was much lower in the bottom layer (Fig 6D), most likely due to sulfate limitation by diffusion [45].

To confirm sulfate limitation at the biofilm bottom layer due to molecular diffusion from the bulk water phase, the maximum penetration depth for sulfate (z_max_ (SO_4_^2-^)) was calculated for the mean sulfate concentration of 2.5 mM observed *in situ* [21] and for 10 mM sulfate, concentration at which biofilm net sulfide production rate was saturated. When sulfate was 2.5 mM or lower, the microaerophilic conditions prevailing in the upper biofilm layer resulted in low net H_2_S production (0.8 μmol H_2_S cm^-3^ h^-1^) (Fig 1). Therefore, sulfate diffused deeper in the biofilm due to a lower sulfate reduction rate in the upper layer, being consumed from a depth of 0.5 mm to the maximum penetration depth of sulfate, 1.3 mm. This agrees well with the shape and modeling results of sulfide profiles (see Figs 1 and 2). However, when the concentration of sulfate in the bulk water phase was increased to 10 mM, both the volumetric net sulfide production rate and the z_max_ (SO_4_^2-^) increased (1.34 μmol H_2_S cm^-3^ h^-1^, and 1.9 mm, respectively), despite the higher sulfate reduction rate.

The upper net sulfide producing layer split into two new layers when nitrate was added to the artificial wastewater: an upper layer consuming sulfide and a middle layer producing sulfide, in some cases, at even a higher rate than without nitrate. Below these layers, the bottom net sulfide producing layer remained basically unaffected. The thickness of the upper net H_2_S oxidizing layer was probably determined by the depth of nitrate penetration within the biofilm.

The addition of 1 mM nitrate generated an upper layer in the biofilm with an average sulfide re-oxidation rate of 1.2 μmol cm^-3^ h^-1^ (Fig 6C and 6F). In this layer sulfide was also oxidized with O_2_ at a rate of 0.264 μmol cm^-3^ h^-1^ and extended down to 0.370 mm depth. Therefore, the difference between total sulfide oxidation rate and O_2_ consumption rate is considered as the sulfide oxidation rate dependent on nitrate (0.936 μmol cm^-3^ h^-1^). Such a rate would result in an estimated maximum nitrate penetration depth of 0.4 mm, which agrees well with the observed maximum depth of the upper net sulfide oxidation layer. Similar penetration depth for nitrate was observed in a sewer biofilm reactor producing sulfide and methane. In this case the nitrate inhibition of methanogenic activity at the biofilm deepest layer was limited by the nitrate penetration depth [14].

In the absence of added nitrate, both biofilm layers followed different trends regarding pH changes with depth. In the upper layer, pH decreased with depth down to the oxic-anoxic interface (0.2 units from pH 6.7 in the water column), likely due to H^+^ production from H_2_S oxidation, either with O_2_ or with NO_3_^-^ [42, 43, 45]. Below the oxic-anoxic interface, H^+^ consumption processes like sulfate reduction seemed to dominate leading to an steady pH increase toward the biofilm bottom. The addition of nitrate apparently did not change the general two-layer pattern of pH profiles (Figs 1 and 6).

### Kinetics of sulfide production in biofilms

Net sulfide production and areal sulfide concentration within the biofilm depended on water phase sulfate, following a Michaelis-Menten kinetics (Fig 2C and 2D). The half saturation constant, K_s,_ was about 1 mM sulfate in both cases, two to three orders of magnitude higher than previously reported from cultures of aquatic SRB species and communities and homogenized biofilms [50–52]. The higher K_s_ reported here is probably due to the fact that our estimated K_s_ integrates the affinity of SRB cells for sulfate plus the diffusional resistance to sulfate mass transfer through the diffusive boundary layer of the biofilm-water interface and within the biofilm. Consequently, our K_s_ is largely dependent on the biofilm thickness and the diffusion coefficient of sulfate within the biofilm, which is typically 60–100% of that in pure water [53, 54]. Sulfate reduction was mainly limited by sulfate availability within the biofilm which in turn depends on sulfate water phase concentration and biofilm thickness. The thicker the biofilm, the higher the concentration of sulfate required to reach the bottom of the biofilm is. Net sulfide production increased until a maximum rate of 0.227 μmol H_2_S cm^-2^ h^-1^ under non limiting sulfate concentration in the bulk water phase (6–8 mM SO_4_^2-^) (Figs 2 and 5).

The biofilms used in this study were grown *in situ* at the Guadalete WWTP, where the sulfate concentration in the influent wastewater is 2.5 ± 0.8 mM (mean for 2009–2011, unpublished results and [21]). This concentration is twice the K_s_ estimated here but about one third of the sulfate concentration at which net sulfide production in biofilms from this WWTP is saturated. This kinetic study revealed that the biofilm net sulfide production rate at the WWTP is limited by sulfate and therefore net sulfide production *in situ* would respond quickly to any increase of sulfate in the inflow wastewater. The limitation of sulfate, the dependence of sulfate diffusion coefficient on temperature [50, 55], and the relatively high Q_10_ (2.2–3.5) of SRB activity in different environments [50, 56–59] explain the large peaks in sulfide production detected during a continuous monitoring of sulfide concentrations *in situ* and its considerable temporal variability [21].

### Sulfide oxidation: the effect of nitrate and sulfate

The addition of nitrate resulted in the rapid reduction of sulfide concentration (Fig 4). Modeling of H_2_S profiles clearly indicated an increase of sulfide oxidation in the upper layer of the biofilms rather than a competitive inhibition of sulfate reduction by heterotrophic nitrate reducers (Fig 6E and 6F). The quick response to nitrate addition suggests the presence of a nitrate limited NR-SOB community within the biofilm able to respond immediately to an increased NO_3_^-^ availability. This type of indigenous NR-SOB communities have been shown to be important for the control of net sulfide production in whole-scale WWTP and biofilms grown in experimental bioreactors [4, 5].

Usually, nitrate addition to waste water systems does not affect sulfate reduction rates, although species composition of the SRB community may change [5, 9, 60]. The syntrophic relationship between SRB and NR-SOB is complex and only some SRB species have the ability to engage in such a relationship. Tolerance to nitrite seems to be a key selective factor for SRB to be able to survive in the presence of NR-SOB [61, 62]. Mass balance calculation in this experiment indicated that the production of nitrite was linearly dependent on the added nitrate concentration and therefore the SRB community was likely affected (Fig 9A and 9B).

Net sulfide oxidation rates estimated according to Eq 4 ranged from 0.05 to 0.72 μmol H_2_S cm^-2^ h^-1^, being dependent on added sulfate and nitrate concentrations. Assuming a sulfide oxidation layer thickness between 0.5 to 2 mm, the maximum specific H_2_S oxidation rate would range between 14.5 to 3.6 μmol H_2_S cm^-3^ h^-1^, respectively. These values are in the range of maximum sulfide oxidation rates directly measured in the experiment by biogeochemical modeling of H_2_S profiles, i.e. 16.9 μmol H_2_S cm^-3^ h^-1^, and similar to those measured in other sulfide oxidizing aerobic and microaerophilic biofilms [43, 45, 63]. Although nSOR are affected by sulfate and nitrate concentrations [43, 45, 63], so far no detailed kinetic analysis has been performed at the biofilm level. nSOR measured here depended on nitrate concentration according to a monosubstrate Michaelis-Menten kinetics, producing K_s_ values for nitrate of 0.58 and 0.55 mM, at 2 and 10 mM sulfate, respectively. These K_s_ values obtained at the biofilm level from microsensor measurements are in same range to that obtained at a bioreactor level (K_s_ = 0.63 mM NO_3_^-^, Villahermosa et al. 2013). These K_s_ values are much higher than those reported for cultures and slurry experiments [64, 65] for the same reasons as discussed previously for sulfate; they integrate the affinity of NR-SOB cells for nitrate plus the diffusional resistance to nitrate through the DBL at the biofilm-water interface and within the biofilm. In principle, NR-SOB activity could be approximated by bi-substrate kinetics, given that the reduction of NO_3_^-^ requires electrons from H_2_S. However, our results clearly indicated that increased NO_3_^-^ concentrations reduced H_2_S concentration within the biofilm without an apparent effect on nSOR (Figs 5 and 8). This suggests that 1) H_2_S oxidation by NR-SOB occurs at a higher rate than production of H_2_S by sulfate reduction, and 2) the NR-SOB community seems to have a very low K_s_, i.e. a high affinity for H_2_S, being able to oxidise H_2_S within the biofilm to very low values, favoring a close coupling between sulfate reduction by SRB and sulfide oxidation by NR-SOB. Sulfate reduction is most likely the limiting step in the syntrophic relationship between SRB and NR-SOB. In the experimental set-up used in this study, the activity of SRB was controlled by the addition of SO_4_^2-^, which allows the kinetic analysis of SRB and NR-SOB syntrophy. The increase of sulfate reduction rate in the presence of nitrate could be due to the reduction of the H_2_S concentration within the biofilm by the concomitant enhanced NR-SOB activity, thus alleviating potential inhibitory effects of H_2_S on sulfate reducers [66, 67] and on the biofilm heterotrophic community of fermenters [68], which in turn would provide more suitable organic substrates for SRB. Moreover, the re-oxidation of sulfide to sulfate by NR-SOB would increase the availability of sulfate in the biofilm.

### Mass budgets: net production and consumption rates of nitrate, nitrite, ammonium and sulfate

Mass balance calculations did not show a net consumption of sulfate in the absence of added nitrate (Table 2), despite the increase in sulfide production rates with increasing sulfate concentrations shown by H_2_S microelectrodes. On the contrary, we observed a non-statistically significant net production of sulfate, suggesting that, in the absence of added nitrate, sulfate was being regenerated within the biofilm from the oxidation of H_2_S or S° with oxygen [69]. Moreover, mass budgets calculations support the kinetic control of the SRB and NR-SOB syntrophic relationship as a bi-substrate reaction since the addition of nitrate stimulated the biofilm’s sulfate demand, being highest at 10 mM sulfate (Table 2). In experiments with pure cultures of *Thiomicrospira sp*. CVO and mixed NR-SOB communities, low H_2_S/NO_3_^-^ ratios in the bulk water phase favored the complete oxidation of H_2_S to SO_4_^2-^according to Eq 4, whereas under nitrate limitation, a high H_2_S/NO_3_^-^ ratio leads to partial oxidation of H_2_S to S^o^ according to Eq 5 [70–72].

$$5\;HS^{-}+8\;NO_{3}^{-}+3\;H^{+}\to 5\;SO_{4}^{2-}+4\;N_{2}+4\;H_{2}O$$ (4)

$$5\;HS^{-}+2\;NO_{3}^{-}+7\;H^{+}\to 5\;S^{o}+N_{2}+6\;H_{2}O$$ (5)

However, the microenvironment within a biofilm is more complex with significant changes in the H_2_S/NO_3_^-^ ratio occurring vertically; high nitrate and no H_2_S at the biofilm surface to the inverse situation in deeper layers. Therefore, sulfate regeneration rates would change correspondently with depth within the biofilm.

N-cycling within microaerophilic waste water biofilms involves complex interactions between nitrogen species. NO_3_^-^, NO_2_^-^, NH_4_^+^, and several gaseous species not measured in this study like N_2_O, NO, and N_2,_ can be intermediate and end products of several metabolic pathways in addition to autotrophic denitrification by NR-SOB [73]. Nitrification, heterotrophic denitrification, dissimilatory nitrate reduction to ammonium (DNRA) and anammox have been reported in WWTP biofilms [74]. Therefore, interpretation of nitrogen species mass balance net changes is, to a large extent, speculative. Nevertheless, we observed several consistent patterns. Thus, nitrate net uptake by the biofilm was linearly dependent on added nitrate concentration when sulfate was not limiting (10 mM, Fig 9C), whereas this relationship was weaker when sulfate was limiting (2 mM). These results also agree with a bi-substrate reaction kinetics based on H_2_S microsensor measurements within the biofilm discussed in previous sections.

Net production of nitrite was observed during the incubations even in the absence of added nitrate, when no net consumption of nitrate was found (Fig 9A). Net production of nitrite was linearly related to both sulfate and nitrate concentrations in the water phase, with rates being highest when both compounds were added at their maximum concentrations. NR-SOB produce nitrite with a NO_2_^-^:NO_3_^-^ stoichiometry of 1:1 using sulfide or elemental sulfur [70–72]. However, in our system the NO_2_^-^:NO_3_^-^ ratio was higher than 1. This fact, in conjunction with the observed production of NO_2_^-^ in the absence of added nitrate, indicates that some other microbial processes, in addition to NR-SOB activity, are producing NO_2_^-^ within the biofilm. Although, no information is available on how NR-SOB interact with other important microbial players in the N-cycle, we can reasonable expect that reduction of sulfide levels by NR-SOB may stimulate other aerobic and anaerobic microbial pathways. On the other hand, production of nitrite by NR-SOB inhibits some species of SRB lacking nitrite reductase activity, whereas other SRB able to reduce nitrite to ammonium can maintain a syntrophic relationship with NR-SOB [61, 62]. Changes in the taxonomic composition of the SRB community in the presence of nitrate and NR-SOB activity have been shown in different systems [5, 21]. However in the short experiments reported here, metabolic changes induced by sulfate and nitrate on these biofilms were not reflected as major changes in the wastewater biofilm bacterial communities.

## Supporting Information

S1 Dataset Dataset used to produce Fig 1. (XLSX)

S2 Dataset Dataset used to produce Fig 2. (XLSX)

S3 Dataset Dataset used to produce Fig 3. (XLSX)

S4 Dataset Dataset used to produce Fig 4. (XLSX)

S5 Dataset Dataset used to produce Fig 5. (XLSX)

S6 Dataset Dataset used to produce Fig 6. (XLSX)

S7 Dataset Dataset used to produce Fig 7. (XLSX)

S8 Dataset Dataset used to produce Fig 8. (XLSX)

S9 Dataset Dataset used to produce Fig 9. (XLSX)
